# Arterial stiffness is associated with oxidative stress and endothelial activation among persons with treated HIV in Zambia

**DOI:** 10.4102/sajhivmed.v22i1.1298

**Published:** 2021-10-28

**Authors:** Theresa Chikopela, Fastone Goma, Longa Kaluba, Wilbroad Mutale, Chris Guure, Douglas C. Heimburger, John R. Koethe

**Affiliations:** 1 Department of Physiology, Faculty of Medicine, Lusaka Apex Medical University, Lusaka, Zambia; 2 Department of Physiological Sciences, School of Medicine, University of Zambia, Lusaka, Zambia; 3 School of Medicine, Cavendish University, Lusaka, Zambia; 4 Department of Health Policy and Management, School of Public Health, University of Zambia, Lusaka, Zambia; 5 Department of Internal Medicine, School of Medicine, University of Zambia, Lusaka, Zambia; 6 Department of Biostatistics, School of Public Health, University of Ghana, Legon, Ghana; 7 Vanderbilt Institute for Global Health, Vanderbilt University Medical Center, Nashville, Tennessee, United States of America; 8 Department of Medicine, Vanderbilt University Medical Center, Nashville, Tennessee, United States of America; 9 Division of Infectious Diseases, Vanderbilt University Medical Center, Vanderbilt University, Nashville, Tennessee, United States of America

**Keywords:** Oxidative stress, endothelial activation, endothelial dysfunction, arterial stiffness, peroxynitrite

## Abstract

**Background:**

Cardiovascular disease (CVD) prevalence is rising among persons with HIV (PLWH) in sub-Saharan Africa. Oxidative stress and endothelial activation, resulting in reduced vascular compliance, are contributors to CVD risk. However, there is a paucity of vascular health data in this population.

**Objectives:**

To assess the relationships of oxidative stress and endothelial activation with vascular stiffness among PLWH.

**Method:**

Fifty-four PLWH on antiretroviral therapy > 5 years and 57 HIV-negative controls, all aged 18–45 years, were enrolled from the University Teaching Hospital, Lusaka, Zambia. Oxidative stress was measured by nitrotyrosine, a peroxynitrite biomarker, and endothelial activation by soluble intercellular adhesion molecule-1 (sICAM-1) plasma levels. Vascular compliance was measured using carotid-radial pulse wave velocity (crPWV) and arterial stiffness index (crASI).

**Results:**

PLWH had higher sICAM-1 levels (median 345 ng/mL) compared to controls (275 ng/mL, *p* < 0.01), as well as higher nitrotyrosine levels (297 versus 182 nM; *p* = 0.02). Median crPWV was similar between the groups, but PLWH had higher crASI (2.4 versus 2.2 cm/ms; *p* < 0.05). After adjusting for age, fat mass, and blood pressure, the estimated effect of a one unit increase in nitrotyrosine on crPWV were twofold higher in the PLWH, but neither reached significance. In a model pooling all participants, there were significant differences in the relationship of nitrotyrosine with crPWV and crASI by HIV status.

**Conclusion:**

PLWH in sub-Saharan Africa had significantly greater oxidative stress and endothelial activation compared to HIV-negative individuals. These factors may contribute to increased arterial stiffness and higher CVD prevalence in this population.

## Introduction

Globally, cardiovascular disease (CVD) is a leading cause of mortality and is estimated to comprise 54% of total deaths.^1^ The prevalence of CVD is on the rise in sub-Saharan Africa (SSA)^2,3^ and overlaps with a persistent epidemic of HIV infection.^4^ Evidence from high-income countries suggests that the incidence of CVD events in persons living with HIV (PLWH) is approximately 1.5–2.0-fold higher compared to age-matched HIV-negative individuals,^5,6^ and PLWH have a significantly higher risk of acute myocardial infarction (MI) compared with demographically and behaviourally similar HIV-negative individuals even after adjustment for Framingham risk factors, comorbidities and substance use.^7,8,9,10^ Notably, this risk differential persists despite suppression of plasma viraemia by antiretroviral treatment (ART).^4,7^

At present, the vast majority of studies of CVD in PLWH are from cohorts in the United States of America (USA) and Europe,^11^ and there is a critical need to understand the factors contributing to CVD among PLWH in SSA, the region with the highest burden of HIV (comprising 61% of PLWH worldwide).^12^

Persistent activation of the endothelium contributes to impaired vasodilatory properties, increased arterial stiffness and disrupted anticoagulatory pathways.^13^ These factors contribute to the development of CVD in the general population^14^; they are heightened or increased in the setting of HIV infection and persist despite plasma viral suppression.^15,16,17^ Upon stimulation, the endothelium becomes activated, and there is upregulation of adhesion molecule expression, including soluble intercellular adhesion molecule 1 (sICAM-1). It has been observed that endothelial cell activation could also lead to endothelial dysfunction.^18,19^ Vascular tone is determined by many competing vasoconstrictor and vasodilator influences, as well as endothelial signalling factors such as nitric oxide. These are important regulators of arterial responsiveness. Among PLWH, there is increased expression of calcium-independent nitric oxide synthase, known as inducible nitric oxide synthase (iNOS); iNOS is induced in response to increased signalling molecules, for example pro-inflammatory cytokines.^20^

In addition to higher levels of oxidative stress, PLWH have higher levels of free radicals, such as superoxide anion (O_2_-).^21^ There is considerable evidence that the vascular production of O_2_- is increased in hypercholesterolaemia, diabetes mellitus, hypertension and cigarette use.^22,23,24,25^ The O_2_- and nitric oxide react to produce the oxidant peroxynitrite.^26,27^ Peroxynitrite transits through cell membranes, in part through anion channels, and promotes DNA fragmentation, resulting in cellular dysfunction.^28^

Peroxynitrite affects both the structure and function of the endothelium^26,28,29,30^ and can trigger apoptosis and poly (adenosine diphosphate-ribose) polymerase (PARP)–dependent cell death.^31^ Peroxynitrite oxidises nitrates to form nitrotyrosine, which is the preferred biomarker for assessing plasma peroxynitrite levels, as observed by Maslov et al.^32^ These effects lead to a loss of endothelial response, which produces several observable changes in function, such as reduced vascular compliance and dilation,^33,34^ resulting in increased arterial stiffness, which can be assessed in the clinical setting by measurement of the pulse wave velocity (PWV). Increased PWV has been reported among PLWH aged > 18 years compared to HIV-negative controls in a study in Cameroon,^35^ as well as Uganda.^36^

The prevalence of CVD is high among PLWH in Zambia,^37^ including lean individuals, unlike much of the evidence from the USA and Europe and among individuals with generally higher body mass indexes (BMIs). Given the rising rates of CVD among PLWH in SSA, it is important to understand endothelial dysfunction, a putative contributory mechanism. There are very few data on oxidative stress and vascular compliance or endothelial activation among PLWH in SSA. To address this research gap, we assessed the relationships of oxidative stress and endothelial activation with vascular function among persons living with and without HIV in Zambia.

## Methods

We conducted a cross-sectional study among adults with and without HIV recruited from the University Teaching Hospital (UTH), an academic tertiary-care centre in Lusaka, Zambia, between September 2018 and June 2019. All participants with HIV were on an ART regimen of efavirenz, tenofovir and emtricitabine (i.e. the combination pill Atripla) for at least 5 years before enrolment. Plasma HIV-1 RNA testing was not available for routine care, but all participants met the clinical criteria for presumed viral suppression, including the absence of clinical disease progression, as well as reconstituted and stable CD4 cell counts. Participants without HIV were recruited from among the individuals who accompanied the PLWH to the ART clinic and from the UTH outpatient medicine department. They were confirmed negative for HIV using rapid tests (Uni-Gold HIV rapid test).

We excluded persons with a prior diagnosis of hypertension, MI, heart failure, any CVD, elevated cholesterol, diabetes mellitus, respiratory diseases such as bronchitis and asthma, pregnant women, tobacco smokers and those with any self-reported acute infections. Information on non-communicable disease risk factors and general sociodemographic data were collected using the World Health Organization (WHO) STEPS survey instrument.^38^ Height and weight were measured using a Micro T3 PW-BMI digital physician scale. Blood pressure was measured in sets of three using an HEM-757 (Omron, Kyoto, Japan) blood pressure–measuring machine.

Enrolment was stratified by BMI to obtain a relatively uniform distribution of underweight (< 18.5 kg/m^2^), normal weight (18.5 kg/m^2^ – 24.9 kg/m^2^) and overweight (≥ 25.0 kg/m^2^) participants

### Plasma biomarkers

All participants fasted for at least 8 h. Blood was collected in ethylene-diamine-tetra-acetic acid (EDTA) tubes and spun at 2700 rpm for 15 min before storing plasma at −80 °C. The endothelial activation marker sICAM-1 was measured using an enzyme-linked immunosorbent assay (ELISA; RAB0219 human sICAM-1 ELISA kit, Sigma Aldrich, Burlington, MA, United States). To measure oxidative stress, nitrotyrosine, a surrogate measure of plasma peroxynitrite, was also measured by ELISA (ab210603; Abcam, Cambridge, Massachusetts, United States [US]). The absorbance of both ELISA kits was read at 450 nm wavelengths using a 96-well microplate reader (BioTek model EL800, BioTek Instruments, Winooski, VT, United States, US).

### Body composition

Waist circumference and hip circumference were measured using flexible tape. The waist–hip ratio was calculated using the two values. Body fat mass was estimated using bioelectrical impedance analysis (Tanita body fat monitor, Tokyo, Japan).^39,40^ This method provides a detailed full-body and segmental-body composition analysis, including information on the weight, body fat percentage, body fat mass, BMI, fat free mass, estimated muscle mass, total body water and basal metabolic rate for the entire body, using bioelectrical impedance analysis or direct-segmental bioelectrical impedance analysis technology.

### Endothelial function measurement

To assess arterial stiffness, we used the carotid–radial pulse wave velocity (crPWV), a measure of arterial stiffness for muscular arteries and an indicator of early alterations in peripheral vascular dynamics.^41^

The crPWV and carotid–radial arterial stiffness index (crASI) were obtained using the Complior analyse unit (V1.9 beta version 2013; ALAM-Medical, Saint-Quentin-Fallavier, France) according to the manufacturer’s protocol. Participants rested for at least 5 min prior to measurement. The carotid probe was gently placed on the neck at the point where the carotid pulse was most palpable. The radial artery probe was applied to the radial artery and adjusted to obtain the best signal. Pressure signals from the sensors were displayed on the screen of the Complior analysis unit to obtain the crPWV, heart rate and transit time. This was repeated two more times, and the mean of three readings was included in the analysis.

## Statistical analysis

Demographic, clinical and oxidative stress variables, sICAM-1, crPWV and arterial stiffness index (ASI) were compared by HIV status using Wilcoxon rank-sum and chi-square tests (STATA V15.0). Continuous variables were expressed as medians (interquartile ranges). Multiple linear regression models were used to assess the relationships between the independent variable (nitrotyrosine) and the dependent variables (crPWV and crASI); these models were adjusted for BMI, blood pressure and age in both HIV+ and HIV– groups because of the sample size. The pooled data (HIV+ and HIV– groups) were adjusted for age, sex, body fat per cent and haemodynamic measures of pulse, heart rate and systolic blood pressure (SBP).

We included an interaction term between HIV status and nitrotyrosine level in the pooled models to assess whether the relationship of nitrotyrosine with the outcomes differed by HIV status. The covariates were selected *a priori* based on the published risk factors for arterial stiffness, including age, sex, body fat per cent and the haemodynamic measures of pulse, heart rate, SBP and diastolic blood pressure (DBP).^42^ The association models were constructed stepwise, adjusting for variables to note which model had the best associative relationship with the outcome variable. The models with the highest *R*^2^ (the proportion of the variance for a dependent variable that is explained by an independent variable) and lowest Akaike information criterion selected for final interpretation. Partial effect plots were also constructed to assess the relationship of nitrotyrosine with arterial stiffness by sex. The normality of the outcome data was assessed using Q–Q plots and validated using the Shapiro–Wilk test. Neither required transformation.

### Ethical considerations

All participants gave written informed consent. Ethical approval for the study was obtained from the University of Zambia Biomedical Research Ethics Committee (UNZABREC – IRB00001131 of IORG0000774) and Zambia’s National Health Research Authority (NHRA).

## Results

We recruited 111 participants, 54 (36 female; 18 male) with HIV and 57 (37 female; 20 male) without HIV, between 18 and 45 years old (Table 1). The participants with HIV were older (*p* < 0.001) and had lower body weight (*p* = 0.04). The medians for most of the other clinical characteristics were comparable.

**TABLE 1:** Clinical characteristics comparing persons living with HIV and the control (HIV-negative persons).

Variable	PLWH				HIV negative				*P*
	*n*	%	Median	IQR	*n*	%	Median	IQR	
Population size	54	49	-	-	57	51	-	-	-
Sex (female)	36	67	-	-	37	65	-	-	0.85
Age (years)	-	-	40	20–45	-	-	24	18–43	< 0.001*
Weight (kg)	-	-	54.8	49.1–69.2	-	-	60.5	53.2–77.9	0.04
Length (cm)	-	-	164	158–169	-	-	162	158–172	0.77
BMI (kg/m^2^)	-	-	20.4	17.8–26.8	-	-	22.8	18.4–28.3	0.11
Trunk fat (%)	-	-	20.1	11.8–28.2	-	-	21.2	12.1–32.2	0.68
Body fat per cent (Tanita) (%)	-	-	23.3	15.0–31.7	-	-	23.3	14.1–34	0.75
Total body fat mass (Tanita) (kg)	-	-	12.3	7.1–23.1	-	-	14.4	7.0–25.7	0.50
Total body fat mass (BodPod) (kg)	-	-	12.0	9.2–25.5	-	-	14.0	6.6–29.2	0.75
Waist–hip ratio	-	-	0.81	0.81–0.90	-	-	0.798	0.70–0.80	0.02*
Central SBP (mmHg)	-	-	113	103–121	-	-	112	102–124	0.65
SBP (mmHg)	-	-	110	103–123	-	-	115	110–123	0.06
DBP (mmHg)	-	-	80.0	74–87	-	-	79.8	75–87	0.84
Mean arterial pressure (mmHg)	-	-	93	86–101	-	-	90	87–98	0.48
Pulse (bpm)	-	-	78	69–85	-	-	73	65–80	0.03*
Alcohol consumption (yes)	30	-	-	-	24	-	-	-	0.16
crPWV (m/s)	-	-	10	9–11	-	-	10	8–11	0.19
crASI (cm/ms)	-	-	2.4	2.1–2.7	-	-	2.2	1.8–2.5	0.048*
sICAM-1 (ng/mL)	-	-	345	252–412	-	-	275	164–350	< 0.01*
Nitrotyrosine (nM)	-	-	297	171–558	-	-	182	156–290	0.02*

* , *P*-values < 0.05.

IQR, interquartile range; BMI, body mass index; SBP, systolic blood pressure; DBP, diastolic blood pressure; PLWH, persons living with HIV; crPWV, carotid–radial pulse wave velocity; crASI, carotid–radial arterial stiffness index; sICAM-1, soluble intercellular adhesion molecule 1.

The two cohorts had comparable medians of crPWV, but a higher crASI of 2.4 cm/ms (interquartile range [IQR] 2.1 cm/ms – 2.7 cm/ms) was observed among PLWH vs 2.2 cm/ms (IQR 1.8 cm/ms – 2.5 cm/ms) (*p* < 0.05) compared to HIV-negative participants. PLWH had higher levels of nitrotyrosine (297 nM [IQR 171 nM – 558 nM] vs 182 nM [IQR 156 nM – 290 nM]; *p* = 0.02) as well as sICAM-1 (345 nM [IQR 252 nM – 412 nM] vs 275 nM [IQR 164 nM – 350 nM]; *p* < 0.01) compared to uninfected controls.

## Association between endothelial dysfunction and oxidative stress

There was no significant association between nitrotyrosine level and crPWV or crASI in the HIV-positive or HIV-negative groups, as highlighted in Table 2. We found that BMI and SBP were associated with both arterial stiffness outcomes in the PLWH. Among the HIV-negative participants, only age was associated with arterial stiffness, as summarised in Table 2.

**TABLE 2:** Multiple linear regression analyses of arterial stiffness and oxidative stress.

Outcome variable	PLWH			HIV negative		
	Coef	CI	*P*	Coef	CI	*P*
**crPWV as outcome variable**						
Age	0.055	−0.030 to 0.140	0.197	0.086	0.002 to 0.171	0.046*
Nitrotyrosine	−0.014	−0.036 to 0.009	0.225	−0.007	−0.036 to 0.021	0.614
BMI	−0.093	−0.185 to 0.002	0.046*	−0.052	−0.014 to 0.031	0.215
SBP	0.052	0.018 to 0.086	0.003*	0.019	−0.013 to 0.051	0.237
**crASI as outcome variable**						
Age	0.011	−0.010 to 0.033	0.290	0.021	0.005 to 0.038	0.013*
Nitrotyrosine	−0.003	−0.009 to 0.002	0.270	−0.003	−0.009 to 0.003	0.288
BMI	−0.047	−0.070 to –0.023	< 0.0001*	−0.014	−0.031 to 0.002	0.086
SBP	0.013	0.005 to 0.022	0.003*	0.004	−0.002 to 0.011	0.180

* , *P*-values < 0.05.

Note: Model adjusted for age, SBP and BMI in both groups.

PLWH, persons living with HIV; CI, confidence interval; coef, β coefficient; crPWV, carotid–radial pulse wave velocity; crASI, carotid–radial arterial stiffness index; BMI, body mass index; SBP, systolic blood pressure.

## Association between endothelial dysfunction and oxidative stress in pooled data of HIV groups

The relationships between oxidative stress and outcomes of endothelial dysfunction as measured by PWV and the ASI differed between the HIV-negative participants and PLWH. Comparisons among the models constructed were evaluated using AIC and coefficient of determination (*R*^2^) values to determine the best performing model. No significant associations were observed using the linear regression models (non-splined), with or without the interaction terms (Appendix 1: Tables 1-A1 and 2-A1). At a glance, the relationships in the model included some that were not straight enough for linear regression, as shown in the *df*beta analysis in the Appendix 1 data (Figure 1-A1). There were apparent bends, clumps and outliers in some plots. It appeared that examination of the multiple regression model using splines may give a better inference of the outcome. Models 4 and 8 (with splines) for crPWV and crASI, respectively, were therefore selected in this study, as they performed better at showing the relationship between the predictive variables and the outcome variables. All interpretations were based on these models. After adjusting for age, sex, waist circumference, hip circumference, per cent body fat, SBP, DBP and HIV status, the associations of nitrotyrosine levels with crPWV and crASI as the outcomes were as shown in Table 3.

**TABLE 3:** Multiple linear regression analyses of endothelial function and oxidative stress.

Outcome variable	crPWV			crASI		
	Coef	CI	*P*	Coef	CI	*P*
Sex (male)	0.444	−0.347 to 1.236	0.271	0.053	−0.138 to 0.245	0.585
Age	0.063	0.008 to 0.133	0.083	0.018	0.004 to 0.031	0.009
Waist circumference	−0.023	−0.064 to 0.018	0.268	−0.011	−0.022 to 0.000	0.052
Hip circumference	0.022	−0.020 to 0.064	0.310	0.002	−0.008 to 0.013	0.637
Body fat	−0.033	−0.080 to 0.015	0.180	−0.005	−0.018 to 0.008	0.465
SBP	−0.028	−0.064 to 0.007	0.118	−0.008	−0.016 to 0.001	0.102
**DBP**	0.121	0.062 to 0.180	0.000*	0.032	0.017 to 0.046	0.000*
rcs_Nitrotyrosine_1	0.122	0.101 to 0234	0.032*	0.029	−0.001 to 0.058	0.107
rcs_Nitrotyrosine_2	−0.213	−0.463 to 0.038	0.096	−0.053	−0.123 to 0.017	0.146
rcs_Nitrotyrosine_3	0.472	−0.308 to 1.252	0.236	0.120	−0.102 to 0.343	0.287
HIV status (positive)	3.866	0.403 to 7.328	0.029*	0.885	0.115 to 1.656	0.024*
HIV interaction rcs_Nitrotyrosine_1	−0.196	−0.354 to –0.039	0.014*	−0.044	−0.082 to –0.006	0.024*
HIV interaction rcs_Nitrotyrosine_2	0.393	0.055 to 0.730	0.023*	0.083	−0.02 to 0.187	0.113
HIV interaction rcs_Nitrotyrosine_3	−1.072	−2.065 to –0.078	0.035*	−0.215	−0.561 to 0.132	0.225
Constant	−0.393	−3.879 to 3.092	0.825	0.293	−1.233 to 1.818	0.707
*R* ^2^	0.356	-	-	0.428	-	-
*F*-test	3.627	-	-	4.872	-	-
AIC	437.620	-	-	124.220	-	-
Number of observations	107	-	-	106	-	-
Prob > *F*	0.000	-	-	0.000	-	-
BIC	477.720	-	-	164.180	-	-

* , *P*-values < 0.05.

crPWV, carotid–radial pulse wave velocity; crASI, carotid–radial arterial stiffness index; DBP, diastolic blood pressure; SBP, systolic blood pressure; rcs, restricted cubic spline; var, variable; HIV interaction rcs_Nitrotyrosine, the interaction between HIV status and nitrotyrosine levels with restricted cubic spline with 3 knots; sex interaction rcs_Nitrotyrosine, the interaction between sex and nitrotyrosine levels with restricted cubic spline with 3 knots; coef, beta coefficient; CI, confidence interval; AIC, Akaike information criterion; Prob, probability; BIC, Bayesian information criterion.

Nitrotyrosine levels were significantly associated with crPWV (β = 0.12; *p* = 0.03). Participants’ HIV status had a significant influence on this relationship, as observed by the significant interaction terms between nitrotyrosine level and HIV status (Table 3). The crASI model showed that HIV status also affected the relationship between nitrotyrosine level and crASI, as illustrated by the significant interaction term between nitrotyrosine level and HIV status in the model (Table 3).

In the partial effect plots, crPWV and crASI values rose with increases in nitrotyrosine levels in all participants (Figure 1). Higher nitrotyrosine levels have been associated with decreased nitric oxide and thus with reduced ability for vasodilation. This could represent a mechanism for the arterial stiffness, as noted.

**FIGURE 1:**
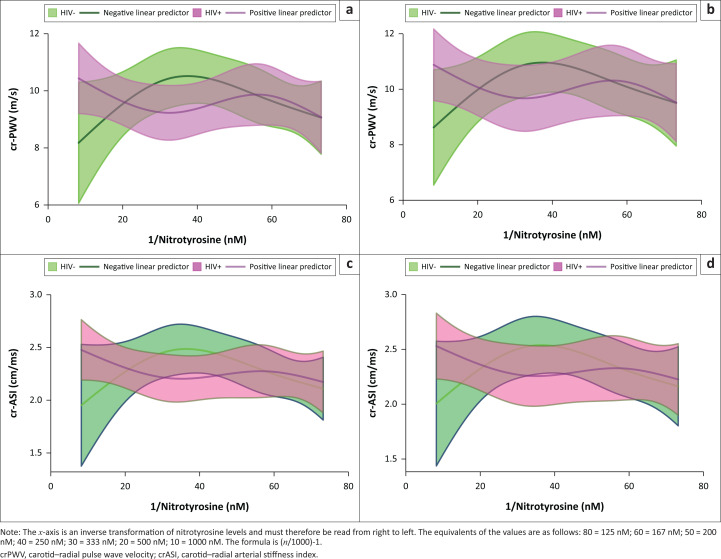
Plasma nitrotyrosine levels predicting endothelial dysfunction (carotid–radial pulse wave velocity [a and b] and carotid–radial arterial stiffness index [c and d]) among individuals without HIV and those with HIV. Other covariates have been set at their medians: age, 32 years old; hip circumference, 93 cm; body fat mass, 24 kg; diastolic blood pressure, 78 mmHg; systolic blood pressure, 121 mmHg; waist circumference, 73 cm. Values for female participants have been plotted on a and c and those for male participants on b and d.

In PLWH with nitrotyrosine levels below 167 nM, nitrotyrosine levels were positively associated with crPWV and crASI, as shown in Figure 1. The crPWV value reached a climax of 10.30 m/s and 9.70 m/s in male and female participants, respectively. The crASI value reached a climax of 2.40 cm/ms and 2.43 cm/ms in male and female participants, respectively. Above 167 nM, an inverse association was observed between nitrotyrosine level and crPWV as well as crASI, until 250 nM. Beyond this, an increase in nitrotyrosine level was positively associated with crPWV and crASI.

Among the HIV-negative participants, nitrotyrosine levels were positively associated with crPWV and crASI below 260 nM, as shown in Figure 1. After this point, there was an inverse association between nitrotyrosine and arterial stiffness. Similar observations were noted for the male and female participants. The arterial stiffness values at any level of plasma nitrotyrosine were higher in male participants than those in the female participants.

## Discussion

**FIGURE 2:**
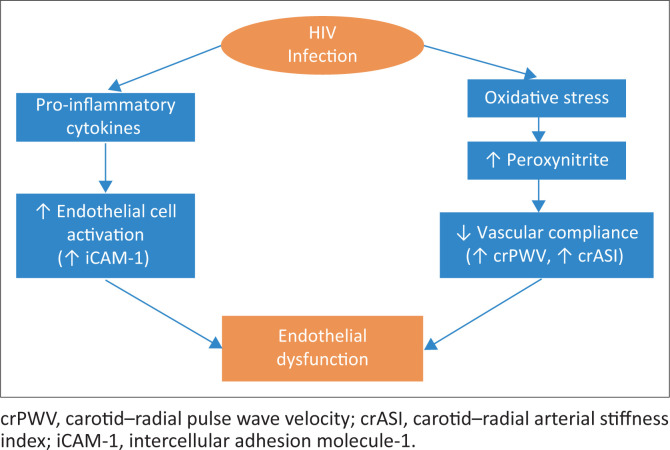
Summary of contribution of endothelial activation and oxidative stress to endothelial dysfunction.

Our cohort of PLWH showed higher oxidative stress compared to the HIV-negative controls, which was associated with increased arterial stiffness. Specifically, we observed that a unit increase in plasma nitrotyrosine was associated with a significant increase in crPWV of 0.12 m/s. This suggests that endothelial dysfunction in PLWH may be associated with oxidative stress-related factors. Nitrotyrosine is a biomarker of peroxynitrite, which causes DNA fragmentation, resulting in cellular dysfunction^28^ and affecting both the structure and function of the endothelium.^26,28,29,30^ This reduces vascular compliance, as conceptualised in Figure 2. Other studies have fortified the idea of peroxynitrite having detrimental effects on the endothelium, which may lead to a disturbance in vascular function.^31,43,44^ Such observations could result from the activity of HIV.^43^ They also observed that ART for 1 month was associated with a significant increase in PWV and ASI in arteries.^45^ Furthermore, increased pulse pressure and augmentation index have been observed among ART-experienced patients compared to untreated PLWH.^46^ Because our study cohort comprised PLWH on stable ART who were assumed to be virally suppressed, endothelial cell damage and the lack of ability to execute its physiological functions could, in part, explain the observations in the PLWH.^26,28^ Our results comparing plasma nitrotyrosine levels in healthy Zambian (black African) populations and PLWH are, as far as we know, the first to be documented. Further, we observed contributory effects of oxidative stress to endothelial dysfunction among PLWH.

Endothelial dysfunction can be measured by the rate of transmission of the arterial pulse pressure wave from the carotid artery, an upstream pressure point, to a defined downstream pressure point (e.g. the radial artery – carotid–radial). The crPWV provides information about the peripheral muscular arteries.

The PLWH in our study had no significant difference in crPWV compared to the HIV-negative participants, implying that virally suppressed PLWH have crPWV values similar to HIV-negative persons. These findings were similar to those of Fourie et al.,^47^ who did not find significant differences in mean crPWV by HIV status. However, Pretorius^48^ observed that crPWV values were significantly higher among PLWH compared to HIV-negative persons (11.26 m/s vs 10.68 m/s; *p* = 0.03) at baseline. And in a follow-up study, after 3 years of ART, no significant differences were found in crPWV values between the two populations.^48^ This supports the idea that virally suppressed PLWH have similar crPWV values to HIV-negative persons after at least 5 years of viral suppression. It may also mean that crPWV is not a good marker for endothelial dysfunction in PLWH.

The ASI is another measure of arterial stiffness quantifying the contour of the oscillometric envelope of peripheral pulse waveform. With increasing arterial stiffness, the arteries become harder to collapse by the application of external pressure. Therefore, with increased arterial stiffness, the oscillometric envelope becomes much flatter. In this study, the crASI, unlike crPWV factors in the participant’s height, was significantly higher among the PLWH (+0.2 cm/ms), implying that there may be an increase in arterial stiffness in this group. Persons living with HIV may have a significantly higher stiffness of the medium, muscular-type arteries.^48^ In the SSA population, crASI seems to be more sensitive in detecting arterial stiffness of the peripheral arteries compared to crPWV. Stiffening of these arteries predominantly results in functional variation, such as increased diastolic blood pressure because of increased muscle tone.^49^ Stiffness in the peripheral muscular arteries is of potential clinical importance.^49^ Peripheral arterial stiffness may be specifically associated with peripheral vascular disease, which is linked to hypertension and diabetes, both of which are risk factors for CVD.^49,50^

We further observed that oxidative stress as measured by plasma nitrotyrosine level was associated with increased arterial stiffness after adjusting for blood pressure, age and body fat mass, in line with the conceptual model in Figure 2. Our models showed that increased oxidative stress is associated with higher arterial stiffness as measured by crPWV. Further, the significant interaction term between HIV and both crPWV and crASI, specifically nitrotyrosine’s significantly different association with crASI and crPWV between PLWH and HIV-negative persons, suggests that the association between oxidative stress and arterial stiffness was influenced by HIV status. Gurovich et al.^51^ also reported an association between plasma nitrotyrosine and PWV in PLWH. We also observed that the male participants had higher arterial stiffness compared to the female participants, possibly suggesting the need for a more robust study to ascertain the predictability of arterial stiffness by nitrotyrosine level stratified by sex.

The endothelial activation marker, sICAM-1, was higher among PLWH. Stimulation of the endothelium results in upregulation of the expression of adhesion molecules, one of which is sICAM-1. The activity of gp120-Tat protein (therefore HIV itself) induces the expression of sICAM-1, which could be a mechanism through which HIV infection contributes to endothelial injury,^52^ as noted in the conceptual model in Figure 2. In our cohort, PLWH had significantly higher sICAM-1 values, consistent with several reports of higher sICAM-1 expressed on vascular endothelial cells in PLWH compared to HIV-negative persons.^53^ Fourie et al.^47^ reported similar observations in their South African cohort, specifically that virally suppressed PLWH had elevated sICAM-1 levels compared to HIV-negative persons.

## Strengths and limitations

The study had several strengths, including the use of HIV-negative controls. The PLWH had a median age of 40 (IQR 33–42) years, older than the HIV-negative participants by 10 years (*p* < 0.001), which could have had an impact on the comparison of arterial stiffness between the groups. We did not measure the viral load to confirm viral suppression but assumed viral suppression based on the length of time participants had been on ART and the absence of clinical disease progression.^54^ The study did not control for cholesterol levels or diabetes, though participants were asked for any history of dyslipidaemia or diabetes. Because of the cross-sectional nature of the study, it is not possible to confirm causality or the mechanisms that might underlie the higher crPWV levels in this cohort. We could not highlight the effects of ART, as the study was not powered to detect such differences. Further, given the observed beneficial effects of ART on vascular function,^16,55^ we could not determine the role that ART played in our cohort, as well as the role of weight gain in individuals on ART. Besides HIV itself, current ART drugs may also play a role in causing oxidative stress, endothelial activation and vascular stiffness. The role of ART in causing these effects needs to be investigated, together with the development of ART drugs with a better ‘cardiovascular’ safety profile. It should be further noted that all participants were on efavirenz, tenofovir and emtricitabine, which is a strength of the study. However, our findings can serve as preliminary characterisations of these variables in PLWH.

## Conclusion

Persons living with HIV in Zambia exhibit increased endothelial dysfunction, as shown by their significantly higher crASI values compared to HIV-negative persons. The PLWH in our study also showed significantly higher endothelial activation markers. The nitrotyrosine levels were elevated among PLWH, signifying an increase in oxidative stress in this group of individuals. Our models revealed that a high nitrotyrosine level was associated with arterial stiffness (crPWV) in a non-linear relationship. Further studies evaluating crPWV, crASI and nitrotyrosine levels from the time of HIV diagnosis will help elucidate how the observed results may vary with time and potentially reveal causal relationships and mechanisms. This will facilitate the individualisation of the treatment of PLWH, considering oxidative stress levels (nitrotyrosine levels) and endothelial activation (sICAM-1) to lower arterial stiffness. There is also a need to determine if these findings are representative of PLWH in Zambia or are more broadly generalisable.

## Figures and Tables

**FIGURE 1-A1:**
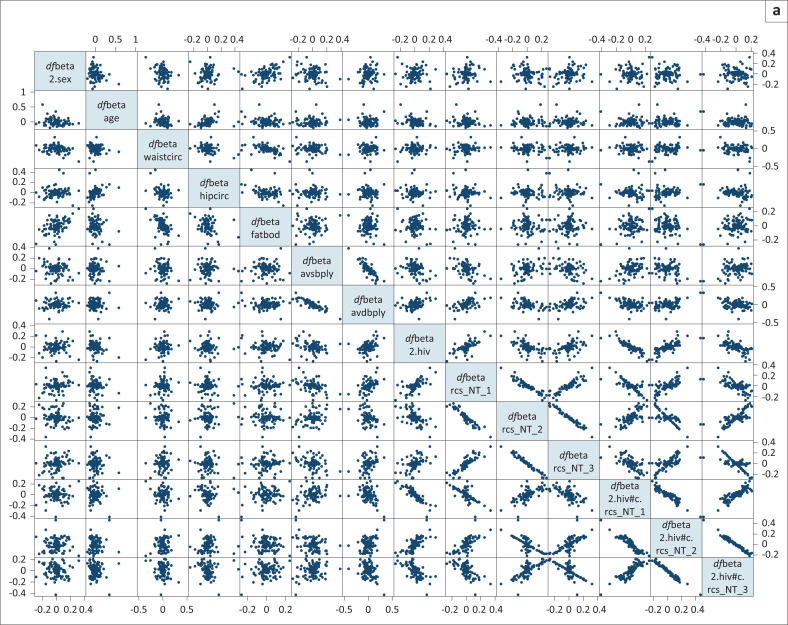

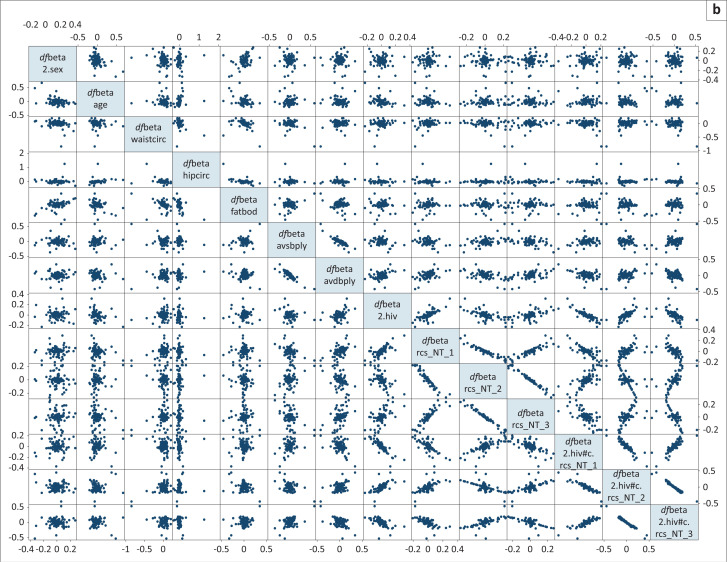
Scatter plot matrix of data showing all possible plots for specified variables with *df*beta for linear regression.

## Appendix 1

### Supplementary material

The following models were used to observe the effect of nitrotyrosine level on crPWV and crASI. All the models include adjustments for age, sex, waist circumference, hip circumference, per cent body fat, SBP, DBP and HIV status. Among the models are one with splines included to note the more fluid (more robust) relationship between the changes in nitrotyrosine levels and those in crPWV or crASI values, as well as one model with the inclusion of an interaction term of nitrotyrosine and HIV status. The final models used to represent these relationships were those showing a higher *R*^2^ value and lower Akaike information criterion (AIC) estimator.

**TABLE 1-A1:** Multiple linear regression analyses of carotid–radial pulse wave velocity and oxidative stress.

Variable	Model 1†			Model 2‡			Model 3§				Model 4¶			
	Coef	CI	F statistic	Coef	CI	F statistic	Coef	CI	*p*	F statistic	Coef	CI	*p*	F statistic
Sex (male)	0.491	−0.479 – 1.461	-	0.508	−0.47 – 1.487	-	0.477	−0.511 – 1.465	-	-	0.444	−0.347 – 1.23	-	-
Age	0.066*	0.008 – 0.124	-	0.065*	0.007 – 0.124	-	0.066*	0.008 – 0.125	-	-	0.063*	0.008 – 0.133	-	-
Waist circumference	−0.027	−0.076 – 0.021	-	−0.028	−0.078 – 0.021	-	−0.027	−0.077 – 0.022	-	-	−0.023	−0.064 – 0.018	-	-
Hip circumference	0.020	−0.018 – 0.058	-	0.020	−0.018 – 0.058	-	0.021	−0.018 – 0.059	-	-	0.022	−0.020 – 0.064	-	-
Body fat	−0.024	−0.082 – 0.034	-	−0.022	−0.081 – 0.036	-	−0.026	−0.085 – 0.033	-	-	−0.033	−0.080 – 0.015	-	-
SBP	−0.019	−0.059 – 0.021	-	−0.019	−0.059 – 0.022	-	−0.017	−0.058 – 0.025	-	-	−0.028	−0.064 – 0.007	-	-
DBP	0.102**	0.037 – 0.168	-	0.102**	0.036 – 0.168	-	0.101**	0.033 – 0.169	-	-	0.121**	0.062 – 0.180	-	-
HIV status	−0.229	−1.174 – 0.716		0.057	−1.775 – 1.889		0.175	−1.716 – 2.067			3.866*	0.403 – 7.328		
Nitrotyrosine	−0.010	−0.026 – 0.007	-	−0.006	−0.033 – 0.021	-			-	-			-	-
rcs_Nitrotyrosine_1	-	-	-			-	−0.009	−0.085 – 0.067	-	-	0.122*	0.101 – 0.234	-	-
rcs_Nitrotyrosine_2	-	-	-			-	0.033	−0.151 – 0.218	-	-	−0.213	−0.463 – 0.038	-	-
rcs_Nitrotyrosine_3	-	-	-			-	−0.200	−0.811 – 0.412	-	-	0.472	−0.308 – 1.252	-	-
HIV interaction nitrotyrosine	-	-	-	−0.006	−0.041 – 0.028	-	−0.007	−0.029 – 0.043	-	-			-	-
HIV interaction rcs_Nitrotyrosine_1	-	-	-	-		-			-	-	−0.196*	−0.354 – –0.039	-	-
HIV interaction rcs_Nitrotyrosine_2	-	-	-	-		-			-	-	0.393*	0.055 – 0.730	-	-
HIV interaction rcs_Nitrotyrosine_3	-	-	-	-		-			-	-	−1.072*	−2.065 – –0.078	-	-
Constant	3.083	−1.052 – 7.219	-	2.989	−1.198 – 7.176	-	2.527	−2.116 – 7.170	-	-	−0.393	−3.879 – 3.092	-	-
Regression details	-	-	F(9, 97) = 4.61	-	-	F(10, 96) = 4.12			< 0.001	F(12, 94) = 3.56		-	< 0.001	F(14,92) = 3.63
***R* ^2^**	0.300	-	-	0.300	-	-	0.313	-	-	-	0.356	-	-	-
***F*-test**	4.601	-	-	4.123	-	-	3.564	-	-	-	3.627	-	-	-
**AIC**	436.56	-	-	438.42	-	-	440.53	-	-	-	437.62	-	-	-
**No. of observations**	107	-	-	107	-	-	107	-	-	-	107	-	-	-
**Prob >*F***	0.000	-	-	0.000	-	-	0.000	-	-	-	0.000	-	-	-
**BIC**	463.294	-	-	467.820	-	-	475.27	-	-	-	477.72	-	-	-

DBP, diastolic blood pressure; SBP, systolic blood pressure; rcs, restricted cubic spline; var, variable; HIV interaction rcs_Nitrotyrosine, the interaction between HIV status and nitrotyrosine levels with restricted cubic spline with 3 knots; sex interaction rcs_Nitrotyrosine, the interaction between sex and nitrotyrosine levels with restricted cubic spline with 3 knots; coef, beta coefficient; CI, confidence interval; AIC, Akaike information criterion; Prob, probability; BIC, Bayesian information criterion.

* , *p* < 0.5;

** , *p* < 0.01.

† , Model 1 was adjusted for age, sex, waist circumference, hip circumference, percentage body fat, SBP, DBP and HIV status;

¶ , Model 2 was adjusted for age, sex, waist circumference, hip circumference, percentage body fat, SBP, DBP and HIV status with an interaction term between HIV status and nitrotyrosine;

§ , Model 3 was adjusted for age, sex, waist circumference, hip circumference, percentage body fat, SBP, DBP and HIV status with an interaction term between HIV status and nitrotyrosine (with splines on nitrotyrosine);

¶ , Model 4 was adjusted for age, sex, waist circumference, hip circumference, percentage body fat, SBP, DBP and HIV status with an interaction term between HIV status and nitrotyrosine (with splines on both nitrotyrosine and interaction term).

**TABLE 2-A1:** Multiple linear regression analyses of carotid-radial arterial stiffness index and oxidative stress.

Variable	Model 5†			Model 6‡			Model 7§			Model 8¶		
	Coef	CI	F statistic	Coef	CI	F statistic	Coef	CI	F statistic	Coef	CI	F statistic
Sex (male)	0.068	−0.155 – 0.292	-	0.068	−0.157 – 0.294	-	0.063	−0.165 – 0.292	-	0.053	−0.138 – 0.245	-
Age	0.019**	0.006 – 0.032	-	0.019**	0.005 – 0.032	-	0.019**	0.005 – 0.033	-	0.018**	**0.004 – 0.031**	-
Waist circumference	–**0.012***	−0.023 – –0.001	-	–**0.012***	−0.023 – –0.001	-	–**0.012***	−0.023 – –0.001	-	−0.011	−0.022 – 0	-
Hip circumference	0.003	−0.006 – 0.011	-	0.003	−0.006 – 0.011	-	0.003	−0.006 – 0.012	-	0.002	−0.008 – 0.013	-
Body fat	−0.004	−0.017 – 0.01	-	−0.004	−0.017 – 0.01	-	−0.004	−0.018 – 0.01	-	−0.005	−0.018 – 0.008	-
SBP	−0.005	−0.015 – 0.004	-	−0.005	−0.015 – 0.004	-	−0.005	−0.015 – 0.004	-	−0.008	−0.016 – 0.001	-
DBP	0.028**	0.012 – 0.043	-	0.028	0.012 – 0.043	-	0.027**	0.012 – 0.043	-	0.032**	0.017 – 0.046	-
HIV status	−0.055	−0.274 – 0.164	-	−0.057	−0.496 – 0.381	-	−0.034	−0.499 – 0.431	-	0.885*	0.115 – 1.656	-
Nitrotyrosine	−0.003	−0.007 – 0.001	-	−0.003	−0.01 – 0.003	-			-			-
rcs_Nitrotyrosine_1	-	-	-	-	-	-	−0.003	−0.021 – 0.015	-	0.029	−0.001 – 0.058	-
rcs_Nitrotyrosine_2	-	-	-	-		-	0.005	−0.039 – 0.049	-	−0.053	−0.123 – 0.017	-
rcs_Nitrotyrosine_3	-	-	-	-	-	-	−0.030	−0.174 – 0.114	-	0.120	−0.102 – 0.343	-
HIV interaction Nitrotyrosine	-	-	-	0.000	0.99 – -0.008	-	0.000	−0.009 – 0.009	-			-
HIV interaction rcs_Nitrotyrosine_1	-	-	-	-	-	-	-	-	-	−**0.044***	−**0.082 – −0.006**	-
HIV interaction rcs_Nitrotyrosine_2	-	-	-	-	-	-	-	-	-	0.083	−0.02 – 0.187	-
HIV interaction rcs_Nitrotyrosine_3	-	-	-	-	-	-	-	-	-	−0.215	−0.561 – 0.132	-
Constant	1.109	0.157 – 2.06	-	1.109	0.143 – 2.075	-	1.036	−0.057 – 2.129	-	0.293	−1.233 – 1.818	-
Regression details		-	F(9, 96) = 6.836		-	F(10, 95) = 6.089	-	-	F(12, 93) = 5.066	-	-	F(14, 91) = 4.872
***R* ^2^**	0.391	-	-	0.391	-	-	0.395	-	-	0.428	-	-
***F*-test**	6.836	-	-	6.089	-	-	5.066	-	-	4.872	-	-
**AIC**	121.020	-	-	123.020	-	-	126.200	-	-	124.220	-	-
**No. of observations**	106	-	-	106	-	-	106	-	-	106	-	-
**Prob >*F***	0.000	-	-	0.000	-	-	0.000	-	-	0.000	-	-
**BIC**	147.660	-	-	152.320	-	-	160.830	-	-	164.180	-	-

DBP, diastolic blood pressure; SBP, systolic blood pressure; rcs, restricted cubic spline; var, variable; HIV interaction rcs_Nitrotyrosine, the interaction between HIV status and nitrotyrosine levels with restricted cubic spline with 3 knots; sex interaction rcs_Nitrotyrosine, the interaction between sex and nitrotyrosine levels with restricted cubic spline with 3 knots; Coef, Beta coefficient; CI, confidence interval; AIC, Akaike information criterion; Prob, probability; BIC, Bayesian information criterion.

* , *p* < 0.5;

** , *p* < 0.01.

† , Model 5 was adjusted for age, sex, waist circumference, hip circumference, percentage body fat, SBP, DBP and HIV status;

¶ , Model 6 was adjusted for age, sex, waist circumference, hip circumference, percentage body fat, SBP, DBP and HIV status with an interaction term between HIV status and nitrotyrosine;

§ , Model 7 was adjusted for age, sex, waist circumference, hip circumference, percentage body fat, SBP, DBP and HIV status with an interaction term between HIV status and nitrotyrosine (with splines on nitrotyrosine);

¶ , Model 8 was adjusted for age, sex, waist circumference, hip circumference, percentage body fat, SBP, DBP and HIV status with an interaction term between HIV status and nitrotyrosine (with splines on both nitrotyrosine and interaction term).
